# Molecular and Genetic Events Determining the Softening of Fleshy Fruits: A Comprehensive Review

**DOI:** 10.3390/ijms232012482

**Published:** 2022-10-18

**Authors:** Zhenzhen Peng, Gangshuai Liu, Hongli Li, Yunxiang Wang, Haiyan Gao, Tomislav Jemrić, Daqi Fu

**Affiliations:** 1 Laboratory of Fruit Biology, College of Food Science and Nutritional Engineering, China Agricultural University, Beijing 100083, China; 2 Institute of Agri-Food Processing and Nutrition, Beijing Academy of Agricultural and Forestry Sciences, Beijing 100097, China; 3 Key Laboratory of Post-Harvest Handing of Fruits, Ministry of Agriculture and Rural Affairs, Food Science Institute, Zhejiang Academy of Agricultural Sciences, Hangzhou 310021, China; 4 Department of Pomology, Division of Horticulture and Landscape Architecture, Faculty of Agriculture, University of Zagreb, 10000 Zagreb, Croatia

**Keywords:** fruit softening, cell wall, phytohormones, transcription factors, epigenetic modifications

## Abstract

Fruit softening that occurs during fruit ripening and postharvest storage determines the fruit quality, shelf life and commercial value and makes fruits more attractive for seed dispersal. In addition, over-softening results in fruit eventual decay, render fruit susceptible to invasion by opportunistic pathogens. Many studies have been conducted to reveal how fruit softens and how to control softening. However, softening is a complex and delicate life process, including physiological, biochemical and metabolic changes, which are closely related to each other and are affected by environmental conditions such as temperature, humidity and light. In this review, the current knowledge regarding fruit softening mechanisms is summarized from cell wall metabolism (cell wall structure changes and cell-wall-degrading enzymes), plant hormones (ETH, ABA, IAA and BR et al.), transcription factors (MADS-Box, AP2/ERF, NAC, MYB and BZR) and epigenetics (DNA methylation, histone demethylation and histone acetylation) and a diagram of the regulatory relationship between these factors is provided. It will provide reference for the cultivation of anti-softening fruits.

## 1. Introduction

Fleshy fruits are important sources of human diet due to their rich nutrients such as carbohydrates, vitamins, trace elements and dietary fiber, which are benefit to the health and immunity of human [1]. They are widely cultivated in the world and loved by most consumers. The commercial value of fleshy fruits is affected by their color, nutritional composition, taste, flavor, texture, etc. Among these, fruit softening plays an important role because it is a sign of fruit full ripening marked with ideal color, flavor and accumulation of a large amount of flavor and nutrients and also is conducive to the dispersion and dissemination of fruit seed; however, excessive softening reduces the resistance of fruits to mechanical injury and pathogen infection, resulting in a serious shortening the shelf life and increasing postharvest losses. [2].

Softening that occurs during fruit ripening and postharvest storage includes comprehensive changes in physiology, biochemistry, cell structure and gene expression [3]. It is not only controlled by internal factors (cell wall metabolism, transcriptional regulation, hormone signal transduction, etc.) but also influenced by external environmental conditions (temperature, humidity, light, etc.) [4]. Extensive studies on softening during the ripening stage and postharvest storage of fleshy fruit have been focused on the model climacteric fruit tomato and the non-climacteric fruit strawberry, as well as on several other economically important fruits such as kiwifruit, banana, peach and grape [5]. Strong evidence demonstrates that the ultrastructure and metabolism of cell wall are key factors during the softening of fleshy fruits [6,7,8,9,10]. Fruit softening is considered a process of cell wall polysaccharide modification or breakdown [5,6]. For example, the CRISPR/Cas9-mediated targeted mutagenesis of tomato polygalacturonase genes (*SlPG*) delays tomato fruit softening [11]. The climacteric fruits require ethylene to start its ripening and softening processes; however, the softening of non-climacteric fruits is more linked to other phytohormones such as abscisic acid and auxin [12,13].

The genetic control of fruit softening increased dramatically by transgenic technology in the last decades [14]. Many transcription factors (LOB1, ERF4, and FSR, etc.) and genes included in phytohormone synthesis and signal transduction (*FIS1*, *SlAREB1*, *SlBES1*, etc.), which are related to fruit softening, have been identified and used to control fruit softening. The silencing of *LOB1*, *FIS1*, *SlBES1*, and *PL* can enhance fruit firmness and prolong fruit’s shelf life without negative effects on tomato fruit flavor and nutrition [15,16,17,18,19,20,21]. Epigenetic regulation including DNA methylation, histone methylation, and acetylation also is reported to be involved in the ripening and softening of many fruits [4]. DNA methylation is located in a cascade with histone methylation modifications [22,23]. In addition, there is increasing evidence that transcriptional factors (TFs), phytohormones, and epigenetic regulators work together to regulate cell wall metabolism during fruit softening [24,25,26,27]. Here, we summarize advances in cell wall metabolism, phytohormones, transcription factors (TFs), and epigenetics related to fruit softening. It will provide a new strategy for improving fleshy fruit quality by controlling fruit softening in the future.

## 2. Cell Wall Metabolism and Structure Remodeling

### 2.1. Cell Wall Structure and Softening-Associated Changes

**Figure 1:**
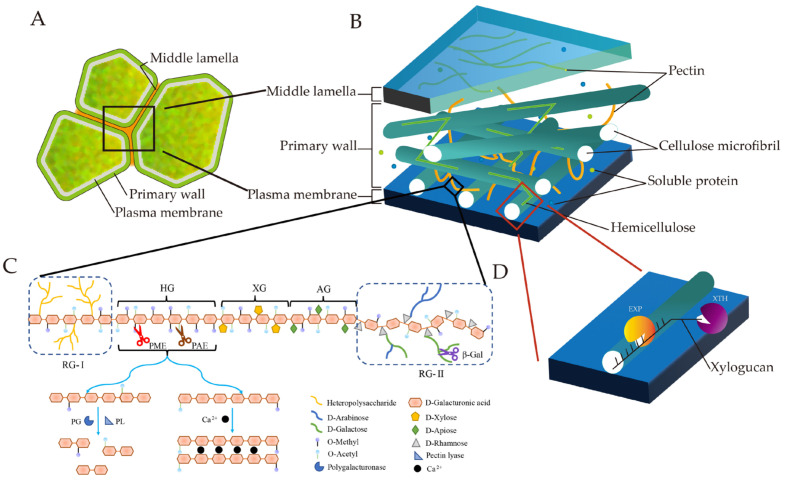
Cell wall structure. (**A**) Schematic representation of the cell wall structure of three cells; (**B**) schematic representation of the structural components of the cell wall (after Farinati et al. [75]); (**C**) schematic representation of the pectin structure. Pectin is composed of five different types of polysaccharides, and various pectin-degrading enzymes are involved in pectin degradation (see text); (**D**) The cross-linking structure of hemicellulose and cellulose microfibril, EXP and XTH are involved in the depolymerization of the structure.

The cell wall of plants not only functions as the first barrier against pathogen attacks but also as the mechanical support of the plant [28]. It is composed of three layers, including the primary wall, middle lamella and secondary wall. The primary wall is mainly composed of cellulose, hemicellulose, pectin, glycoprotein, etc., and is accompanied by the deposition of polysaccharides during cell expansion. The secondary wall characterized by thickening and lignification contains cellulose, hemicellulose, pectin and lignin [29]. The middle lamella responsible for gluing adjacent cells together is comprises pectin between primary walls [30]. The composition of the cell wall determines the cell structure, resulting in different shapes, sizes and functions of the plant cells [31] (Figure 1A). Usually, the cell wall structure of fruits mainly consists of a parenchyma layer, especially in fleshy fruits at the ripening stage, at only the primary wall and the middle lamella and rarely develops to secondary walls [32]. Fruit softening is accompanied by changes in cell wall ultrastructure and cell wall metabolism, which affect fruit texture [8,25]. The cell wall ultrastructure during fruit softening includes the dissolution of the middle lamella and the gradual disintegration of the fibrous matrix throughout the cell wall, resulting in a decrease in cell adhesion, an increase in cell gaps and a decrease in cell wall thickness and density [33,34,35,36]. Fruit softening is mainly influenced by components and metabolism of the cell wall, which involves the coordination of a number of cell-wall-modifying enzymes and proteins. The fruit’s cell wall is mainly composed of cellulose, hemicellulose and pectin. Cellulose and hemicellulose are interwoven by glycosidic bonds and hydrogen bonds, forming a polysaccharide skeleton as the supporting structure of the cell (Figure 1B). The calcium ion and pectin in the fruit cell wall can form a calcium bridge through a covalent bond to mediate adhesion between cells, resulting in enhancements in cell wall strength and fruit firmness [6,37].

### 2.2. Roles of Cell Wall Degrading Enzymes on Fruit Softening

Pectin related to fruit softening is a class of polysaccharides consisting of galacturonic acid, homogalacturonans (HGs), rhamnogalacturonan-I (RG-I), rhamnogalacturonan-II (RG-II), xylogalacturonan (XG) and apiogalacturonan(AG)[35]. Pectin can be degraded into soluble pectin and pectate by demethylation during fruit softening, leading to cell wall loosening and disintegration [36]. In addition, metabolism processes of the cell wall components of fruit occurs during fruit softening, such as hemicellulose dissolution and depolymerization, cell wall polysaccharide degradation and the reduction in the esterification degree of pectin and neutral sugar content in pectin. [38,39]. The degradation enzymes related to cell wall metabolism include pectinase, hemicellulose and cellulase, but non-enzymatic pathways are also a factor correlated with fruit softening [40]. Pectin methylesterase (PME), pectin lyase (PL), polygalacturonase (PG), β-galactosidase (β-Gal), xyloglucan endotransglucosylase/hydrolase (XTH) and expansin (EXP) are the major cell-wall-degrading enzymes synergistically involved in cell wall degradation during fruit softening [10,41]. PME randomly hydrolyzes the methylated carboxyl group in polygalacturonic acid to form pectate, the substrate of PG; however, PME can also act on galacturonic acid by a linear block fashion to produce long stretches of negatively charged carboxylates that cross connect with Ca^2+^, resulting in reduced cell wall porosity and the hardening of the cell wall [42,43]. PL acts on the deesterified polygalacturonic acid to form a double bond between C4 and C5 and finally blocks the α-1,4-glycosidic bond to produce pectin oligosaccharides [44]. PGs are divided into three types: external PGs, internal PGs and rhamnose PGs. Among these, internal PGs participate in the degradation of polygalacturonic acid and pectin in the fruit’s cell walls [45].

In general, the cell wall mainly undergoes the following metabolic reactions related to softening during fruit ripening: PME catalyzes the demethylation of pectin, PL catalyzes the decomposition of deesterified polygalacturonic acid, and PG catalyzes the hydrolysis of α- 1,4-galacturonic acid to produce uronic acid. Fruit softening is dynamically regulated by the above three factors (Figure 1C). The results showed that the silencing of *PE1* or *PMEU1* in tomato accelerates fruit softening, while the co-silencing of *PE2* and *PE1* in tomato would completely prevent ripening [46]. The overexpression of *FvPME38/39* in strawberries reduces fruit firmness by affecting pectin content and cell wall structure, implying that PME plays a key role in the fruit softening process [43,47]. In apricots, the transcript level of the pectin methylesterase gene *PaPME1* elevated with increased storage times [47]. The silencing of *PL* in tomato and strawberry decreases water-soluble pectin (WSP) content and enhances fruit firmness without affecting other aspects of ripening, while the overexpression of *FvPLA* accelerates fruit softening in strawberry fruit [7,48]. Similarly, the silencing of *PpePL1/15* delays softening by inhibiting the depolymerization of pectin in peach fruit [49]. The overexpression of *SlPG49*, a newly identified PG gene in tomato fruit, promoted tomato fruit softening, and the silencing of *FaPG1* in strawberry enhances the fruit’s firmness by inhibiting cell wall degradation and extends its shelf life [50,51]. In other fleshy fruits such as peach, grapevine, banana and pear, it is reported that their fruit softening is also regulated by *PG* genes [52,53].

β-Gal catalyzes the hydrolysis of the β-galactoside side chains into pectin during fruit ripening [54]. The silencing of β-galactosidase genes *SlTBG4* in tomato or *FaβGal4* in strawberry can inhibit fruit softening [55,56]. Genetic analysis in peach revealed that β-gal (encoded by *PpBGAL2* and *PpBGAL16*) is responsible for cell wall degradation during fruit softening [57], and silencing *PpBGAL10* and *PpBGAL16* by virus-induced gene silencing (VIGS) delayed fruit softening of peach [58]. XTHs (xyloglucan endotransglucosylase/hydrolase) catalyses xyloglucan endohydrolysis (XEH) and/or endotransglycosylation (XET) are involved in the modification of cell wall structure by cleaving and, often, are also involved in re-joining xyloglucan molecules in primary plant cell walls [59] (Figure 1D). The transient overexpression of *MdXTHB* gene in ‘Golden Delicious’ and ‘Fuji’ apples promotes apple fruit softening, and the transient overexpression of *FvXTH9* and *FvXTH6* in strawberries resulted in faster fruit ripening [60,61]. In addition, both *DkXTH6* and *DkXTH7* have been shown to play important roles in the softening process of persimmon fruits during storage. *DkXTH6* induces persimmon fruit softening by participating in cell wall recombination and loosening, while *DkXTH7* can maintain the firmness of mature fruit by participating in cell wall synthesis [62]. There is evidence that a reduction in XET activity during ripening may contribute to fruit softening. The overexpression of *SlXTH1* in tomatoes delays fruit softening in transgenic tomatoes [63].

Expansin (EXP), a plant cell wall expansin protein, can break the chemical bond between microfibrils and glucans by binding to glucan-coated cellulose, which makes cellulose’s surface glucans susceptible to cellulases, resulting in fruit cell wall loosening [64] (Figure 1D). EXPs are associated with fruit softening and have different expression patterns in tomato, strawberry, banana and other fleshy fruits [65,66,67]. The silencing of *LeEXP1* in tomatoes enhances fruit firmness and extends its shelf life, while the co-silencing of *LeEXP1* and *LePG* further inhibited fruit softening [68,69]. The expression levels of *PsEXPA10* and *FaEXPA2/5* increased significantly during the softening of plum and strawberry fruits [70,71,72]. In addition, pectin acetylesterase (PAE) is also involved in fruit softening. For example, the overexpression of *MdPAE10* encodes pectin acetylesterase and leads to a significant reduction in flesh firmness. In contrast, silencing *MdPAE10* delays the decline of fruit firmness [73]. *FaEG1* genes encoding endo-β-1, 4-glucanase are reported to be involved in the breakdown of cellulose and hemicellulose structures and play a role in cell wall restructuration during strawberry fruit softening [74]. In summary, the cell wall metabolism related to fruit softening is delicate and complex, including a variety of enzymes related to cell wall metabolism, as shown in Table 1.

## 3. Phytohormones Regulation of Fruit Softening

Phytohormones play an important role in fruit development and ripening. such as ethylene (ETH), abscisic acid (ABA), auxin (IAA), and brassinosteroid (BR), etc. For example, the ripening and softening of climacteric fruits depend on the production of a large amount of ethylene, while non-climacteric fruits depend on ABA to start their fruit ripening and softening processes [76].

### 3.1. ABA

ABA regulates fruit softening in the upstream signaling pathway of ethylene [77]. For example, ABA promotes ETH production to reduce pectinesterase activity, which accelerates fruit pulp softening during mango fruit ripening [78]. The overexpression of the *lycopene β-cyclase* gene (*LCYb*) in tomato results in increased levels of β-carotene (provitamin A), a precursor of ABA. The increased ABA level leads to a reduction in ethylene production; an increase in cell density; the thickening of the fruit’s cuticle; the down-regulation of *PG1, PL* and *EXP1* genes; and the up-regulation of *PMEU1/2*, which finally delays fruit softening and prolongs the fruit’s shelf life [79]. The silencing of *SlNCED1*-encoding 9-cis-epoxycarotenoid dioxygenase (NCED) in the biosynthesis of ABA results in the down-regulation of cell wall metabolism gene (*SlPG*, *SlPME*, *SlTBG*, *SlXET*, *SlCELs* and *SlEXP*) expression and inhibits fruit softening [80]. On the other hand, ABA can induce changes in the transcription level of genes encoding cell-wall-degrading enzymes. For example, exogenous treatments with ABA can accelerate strawberry fruit softening by dramatically promoting the accumulation of transcripts of cell wall metabolism genes such as *FcPG*, *FcRGL1* and *FcEXP5* [81]. Moreover, the overexpression and repression of *SlPYL9*, encoding the ABA receptor, can accelerate or delay fruit softening and pericarp thickness by affecting the transcription of cell wall modification genes (*SlPG* and *SlEXP6*) [82]. Treatments with ABA can cause the postharvest softening of blueberry fruits by inducing the expression of *VcPEs*, *VcPGs*, *VcCx* and *VcGal* [83]. The silencing of *PacCYP707A2*, which encodes the ABA degradation enzyme in sweet cherries, promotes the accumulation of ABA and the up-regulation of *PacNCED1* transcript levels, resulting in the up-regulation of *PacACO1* and a reduction in fruit firmness [84].

### 3.2. ETH

Ethylene is the first-known plant hormone involved in fruit softening. Numerous studies have shown that ETH promotes the softening of climacteric fruits, and the process of fruit softening is influenced by ETH synthesis, distribution, signal transduction and downstream genes. The peak of ethylene production during fruit ripening is necessary for the ripening of climacteric fruit fruits, such as tomatoes, kiwifruit, apricots and plums. For example, exogenous treatments with ETH can rapidly reduce the firmness of kiwifruit and strawberry fruit by inducing *CkPGC* and *FaPG1* gene expressions [85,86]. ETH treatments also accelerated the softening of papaya and apricot fruits due to cell wall decompositions, while ethylene receptor inhibitor (1-methylcyclopropene, 1-MCP) can delay fruit softening by inhibiting the expression of genes related to cell wall degradation [87,88]. The silencing of the genes of key enzymes of system II ethylene biosynthesis in tomatoes, such as *SlACS2*, *SlACS4*, *SlACO1* and *SlACO3*, significantly inhibited fruit softening [89]. ETH receptors are negative regulators of ethylene signal transduction, and it was found that tomato fruit with *LeETR4* or *LeETR6* single mutants exhibited high sensitivity to ETH, and exogenous ETH treatments could rapidly degrade *LeETR4* or *LeETR6* via the 26s protease pathway to regulate tomato ripening [90,91]. The *LeEIN2* gene is a positive regulator of the ETH signaling pathway. The silencing of *LeEIN2* significantly inhibits fruit softening by the down-regulation of softening-related genes (*PG*, *E4* and *TomLoxB*) [92,93].

### 3.3. IAA

In addition, genes encoding DNA demethylases are inhibited by IAA to maintain high methylation levels in the fruit, which inhibits the ripening process [94,95]. *PpYUC11*, a key gene involved in IAA biosynthesis, regulates IAA levels at the later stage of peach ripening and also is a candidate gene for regulating peach fruit softening [96]. *PpIAA1* in peach can bind to the *PpACS1* promoter to activate its expression. The overexpression of *PpIAA1* in tomato accelerates fruit ripening and shortens fruit shelf life by increasing the production of ETH and controlling the expression of ripening-related genes [97]. On the other hand, the overexpression of the IAA-responsive gene *PpSAUR43* down-regulates the cell wall degradation gene *PpPG* and delays the softening of peach fruit [98]. In kiwifruit, the silencing of *AcGH3.1*, a gene controlling IAA homeostasis, increased fruit firmness during ripening and delays fruit softening during postharvest storage. The expression of *AcGH3.1* was also induced by 1-naphthylacetic acid (NAA, IAA analogue) [99]. The application of exogenous IAA in grapes inhibits the accumulation of ABA and suppressed the expression of softening-related genes, such as *PE*, *PG*, *PL* and *CELL*, which delayed fruit ripening and softening [100]. Similarly, exogenous IAA reduced the transcription level of genes encoding pectate lyase, β-D-xylosidase, endoglucanase, β-galactosidase and endo-1,3-β-glucosidase in strawberry and promoted the cellulose-xyloglucan framework’s reorganization by activating the expression of *XT1* and XTH, which delayed the softening of fruits during postharvest storage, [101].

### 3.4. BR

BRs are a group of steroid hormones involved in plant growth and development [102]. Recent studies have shown that BRs have an effect on fruit ripening. For example, exogenous brassinolide treatments of tomato promoted fruit softening by increasing the production of ETH, as did persimmon and banana fruits [103,104,105]. The overexpression of *SlCYP90B3* gene involved in brassinosteroid biosynthesis delays fruit softening by up-regulating ETH biosynthesis (*SlACS2/4* and *SlACO1*) and signaling (*SlETR3* and *SlCTR1*) in tomato fruits. However, silencing *SlCYP90B3* inhibits the softening of tomato fruit [106]. *MaBZR1* and *DkBZR2* involved in BR signaling mediated fruit softening by regulating genes involved in ETH biosynthesis and cell wall degradation in bananas and persimmons. The transient overexpression of *DkBZR2* in persimmons promotes the conversion of acid-soluble pectin to WSP and increases ETH production in fruits [104,107].

### 3.5. Other Phytohormones

**Figure 2:**
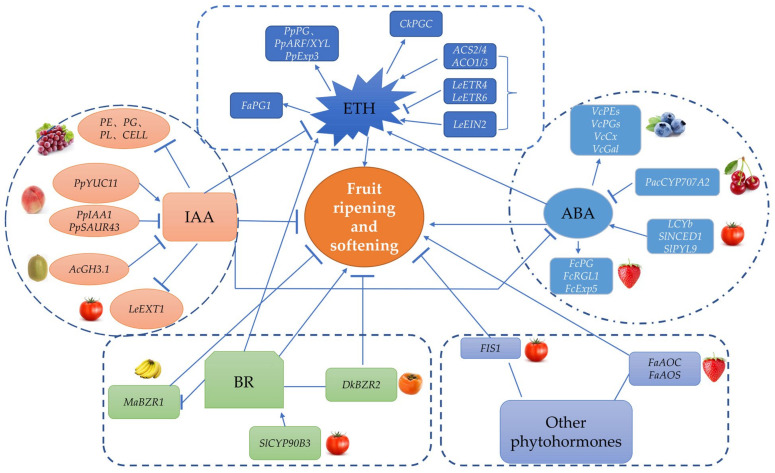
Regulation of ripening and softening of fleshy fruits by phytohormones (see text). It mainly consists of five aspects, including ETH, ABA, IAA, BR and other phytohormones, which are represented by different colors. Arrows indicate activation, while arrows with blunt-ends indicate inhibition, a line means that uncertainty is active or suppressed.

In addition to ETH, ABA, IAA and BRs, gibberellins (GA), jasmonic acid (JA) and salicylic acid (SA) are also involved in fruit softening. A mutant of *FIS1* gene encoding GA2-oxidase increased the level of bioactive gibberellins and enhanced the accumulation of cutin and wax by regulating the expression of cuticle accumulation and deposition-related genes (*CYP86A69*, *CYP77A-LIKE*, *GDSLs* and *CERs*), which increased fruit firmness and shelf life without adverse effects on fruit weight or flavor. It will provide a potential strategy for tomato breeders to cultivate varieties resistant to softening [18]. The exogenous treatment of strawberries and grapes with JA induced fruit softening by promoting the expression of genes involved in cell wall metabolism. The transient overexpression of *FaAOC* and *FaAOS*, two key genes involved in jasmonic acid synthesis, accelerated strawberry fruit softening [108,109]. Treatment with SA effectively delayed fruit firmness decline in tomato by strongly inhibiting the expression of ETH biosynthesis genes (*ACO1* and *ACS2*) and reducing the activity of cell-wall-degrading enzymes [110]. In summary, the softening process is controlled by phytohormones and also involves the co-regulation of other factors (Figure 2). The complex molecular mechanism of phytohormone-regulating fruit softening has not been fully understood, and it needs to be better investigated.

## 4. Transcriptional Regulation of Fruit Softening

Although different tissues of the same organism contain the same genomic DNA, the transcription of each gene is time- and space-specific. For example, tomato fruit is green and hard at the immature stage, whereas it becomes red and soft at the ripening stage due to the spatiotemporal expression specificity of genes related to ripening and softening. The transcription of genes is regulated by transcription factors. Transcription factors can bind to the promoter region of target genes to activate or inhibit their transcriptional factor. Fruit softening occurs during fruit ripening and is regulated by several transcription factors related to fruit softening. Currently, many transcription factors related to softening have been isolated and verified.

### 4.1. MADS-Box TFs and Fruit Softening

RIN (ripening inhibitor) is an important MADS-Box TF involved in all aspects of fruit ripening and directly targets many ripening-related genes (*PG*, *TBG4*, *EXP1*, *CEL2*, etc.). Compared to the wild tomato fruit, the fruit of *ripening inhibitor* (*rin*), a tomato natural ripening mutant, softens slowly and has a long shelf life [110]. SlMBP8, MADS-Box TFs, has also been reported to play an important role in fruit softening. Silencing *SlMBP8* significantly induced the expression of cell wall metabolism genes such as *PG*, *EXP*, *HEX*, *TBG4*, *XTH5* and *XYL* and increased the production of ETH by activating the expression of ETH-related genes, resulting in faster water loss and lower storage tolerance in fruits [111]. FRUITFULL homologs (FULs), MADS-Box TFs, also interact with RIN, and the simultaneous silencing of *FUL1* and *FUL2* delays fruit ripening and reduces the production of ETH and the thickness of the fruit peel’s cuticle, resulting in the inhibition of fruit softening [112,113]. The silencing of *MaMADS1* or *MaMADS2* in banana delays ripening and softening by inhibiting ETH synthesis [114]. The silencing of *PrupeSEP1*, a member of the MADS-box TF subfamily, delays fruit softening in melting flesh peaches, and the expression levels of softening-related genes such as *ACS2*, *EIN2*, *PME1*, *Endo-PG3*, *ACO1*, *ETR2*, *β-Hex2* and *Lox1* were significantly reduced [115]. The silencing of *PrupeFUL4* in peach fruit also delayed fruit softening and significantly suppressed the expression of *PrupACO1* and *PrupACS2*, whereas the overexpression of *PrupeFUL4* accelerated fruit softening by significantly up-regulating *PrupeACO1* [116]. *PaMADS7* activates *PaPG1* expression by directly binding to the *PaPG1* promoter involved in fruit softening in sweet cherries, and the silencing of *PaMADS7* in sweet cherry inhibits fruit softening [117].

### 4.2. AP2/ERF TFs and Fruit Softening

AP2/ERF, a superfamily TFs in plants, is not only involved in fruit ripening but also can regulate the biosynthesis of phytohormones and involved in hormone signaling [118]. AP2a, a member of the AP2/ERF family, negatively regulates ethylene production, and the silencing of *AP2a* in tomato promotes ETH production and fruit softening [119]. The overexpression of *SlERF.B3* genes in tomato accelerates tomato fruit softening by up-regulating the key *PG2a* gene [120]. A recent study showed that *SlERF.F12*, a negative regulator of tomato ripening, forms a triplex with TPL2-HDA1/3, and its overexpression suppresses the expression of ripening-related genes *ACS2*, *ACS4*, *PG2a* and *PL*, which delays fruit softening and prolonging shelf life [121]. PpERF4 in peach activates the transcription of *PpACO1* and *PpIAA1* genes by binding to their promoters. PpIAA1 interacts with PpPRF4 to form a complex, which activates the transcription of ABA biosynthesis genes (*PpNCED2/3*) and fruit-softening genes (*PpPG1*), and ultimately regulates peach fruit ripening and softening by integrating IAA and ETH signals [97]. Recently, Cheng et al. [122] found that PpERF/ABR1 directly binds to the *PpPG* promoter to activate its expression, which also induces peach fruit softening, and the overexpression of *PpERF/ABR1* promotes the production of ETH in accelerating fruit softening. CpERF9 [123], MdERF4 [16] and MaERF11 [124] repress the transcription of cell wall degradation genes during the ripening of papaya, apple and banana.

### 4.3. NAC TFs and Fruit Softening

NAC proteins are involved in a variety of life processes as plant-specific TFs. Several researchers have shown that NAC proteins positively regulate tomato fruit ripening. For example, silencing *SNAC4* in tomatoes maintains fruit firmness and reduces the accumulation of ABA during fruit ripening. SNAC4 directly binds to the promoter regions of *SAPK3*, *SlCYP707A1*, *SlACS8* and *SlACO6* to activate their transcription, which are involved in the synthesis of ABA and ETH and their signal transduction [125]. Gao et al. also found that cell wall degradation genes *SlPG2a*, *SlPL*, *SlCEL2* and *SlEXP1* are targets of *NOR-like1*, which affects fruit softening by regulating pectin solubilization, cellulose-hemicellulose skeleton depolymerization and cell wall loosening [126]. Subsequently, it was found that NOR, a homolog of NOR-like1, can also bind to the promoter of *SlPL* and activate its transcription, and the softening of *nor* mutant fruit is inhibited, while fruit softening is accelerated when *NOR* was overexpressed [127]. The overexpression of another NAC TF, *SlNAC1*, in tomato leads to a reduction in fruit firmness and peel thickness and produces more ABA than wild types [128]. Recently, a mutation of *NOR* by gene editing in melon was found to prevent normal fruit softening, significantly suppressing ETH and ABA production and ultimately extending the shelf life of melon fruits [129]. MaNAC1 and MaNAC2 negatively regulate the ethylene synthesis of banana fruit through MaXB3-mediated ubiquitination degradation pathways, resulting in the inhibition of the softening of banana fruits [130]. FcNAC1, a strawberry fruit ripening-related TF can bind to the *FcPL* promoter to activate its expression, which was involved in pectin metabolism during strawberry fruit softening [131]. Moreover, the silencing of *FaRIF* (*FaNAC035*) enhances fruit firmness by down-regulating the expression of cell wall degradation (*FaXYL3* and *FaPL3/4*) and modification-related genes (*FaEXP1/2/3* and *FaPME39*) [132].

### 4.4. MYB TFs and Fruit Softening

MYB TFs are a superfamily of TFs in plants. MYB TFs numbering 140 and 110 have been identified, respectively, in tomato and strawberry fruits, and some of these play important roles in cell wall’s metabolism, secondary metabolism and stress response [133]. In tomatoes, SlMYB70, a transcription suppressor, can bind to the promoters of *SlACS2 and SlACO3* genes to inhibit their transcription. Silencing *SlMYB70* promotes ethylene production to accelerate fruit ripening and softening [89]. In addition, the overexpression of *SlMYB75* prolongs tomato fruit shelf life by directly down-regulating the expression of *SlFSR*, which regulates the transcription of genes related to cell wall modification [134]. The transient overexpression of *FvMYB79* in strawberry significantly increases the transcriptional level of *FvPME38*, resulting in fruit softening and ripening, while silencing *FvMYB79* delays fruit ripening and increases fruit firmness [135].

### 4.5. BZR TFs and Fruit Softening

The BRASSINAZOLE RESISTANT(BZR) TF is a key component of the brassinosteroid signaling pathway. Recently, SlBES1 in tomato was found to contain the BZR structural domain and to be involved in the fruit softening process. SlBES1 directly binds to the *PMEU1* promoter and inhibits its expression, and the CRISPR-Cas9 knockout of *SlBES1* increases fruit firmness and prolongs its shelf life without affecting fruit nutritional quality [20]. As a transcriptional inhibitor, Banana MaBZR1/2 can directly inhibit ETH biosynthesis and fruit softening by binding to the promoter of ethylene biosynthesis-related genes (*MaACS1*, *MaACO13* and *MaACO14*) and cell wall modification genes (*MaEXP2*, *MaPL2* and *MaXET5*). [136].

### 4.6. Relationship between Other Transcription Factors and Softening

In addition to the ripening-related TFs associated discussed above, silencing *SlFSR*, a member of GRAS TF family TFs, decreases the expression level of several genes related to cell wall modifications, such as *PG*, *TBG*, *CEL* and *XYL*, resulting in extending the shelf life of tomato fruits [17]. The transient overexpression of ARF TF *CpARF2* in papaya decreases fruit firmness, while the transient silencing of *CpARF2* increases fruit firmness, and the ectopic overexpression of *CpARF2* in tomato also decreases fruit firmness and induces the production of ETH in the transgenic lines [137]. SlLOB1, a tomato LATERAL ORGAN BOUNDARIES TF, can activate the transcription of *EXP1*, *CEL2*, *XY*, *AGP2*, *TBL*, *E6* and *PL* gene in vitro, and the overexpression of *SlLOB1* induces cell wall gene expression and promotes fruit softening, while the inhibition of its expression delays fruit softening [15]. The WRKY protein plays a key role in regulating growth, development and plant responses to biological and abiotic stresses. Recently, it was found that the overexpression of *FvWRKY48* in strawberries accelerates fruit ripening and increases the level of high galacturonic acid degradation in the pectin cell wall polymer of the middle lamella and three cell junction regions in the fruit. *FvWRKY48* can bind to the *FvPLA* promoter and promote pectin acid lyase activity, accelerating pectin degradation and fruit softening [48]. In banana, MabZIP74 inhibits the expression of the ETH biosynthesis genes, such as *MaACO1* and *MaACO4* [138]. The expression of bHLH TFs *MaTCP5*, *MaTCP19* and *MaTCP20* were all induced by ETH. MaTCP5 and MaTCP20 are transcriptional activators. *MaTCP19* is a transcriptional repressor. MaTCP5 and MaTCP20 activate the transcription of *MaXTH10*/*11* involved in fruit softening during banana ripening. MaTCP19 represses its transcription by directly binding to the promoter. More importantly, MaTCP20 interacts synergistically or antagonistically with MaTCP5 or MaTCP19 to enhance or attenuate the transcription of *MaXTH10*/*11* [139].

As mentioned above, different types of transcription factors are involved in fruit softening. (Table 2).

## 5. Epigenetic Modifications of Fruit Softening

**Figure 3:**
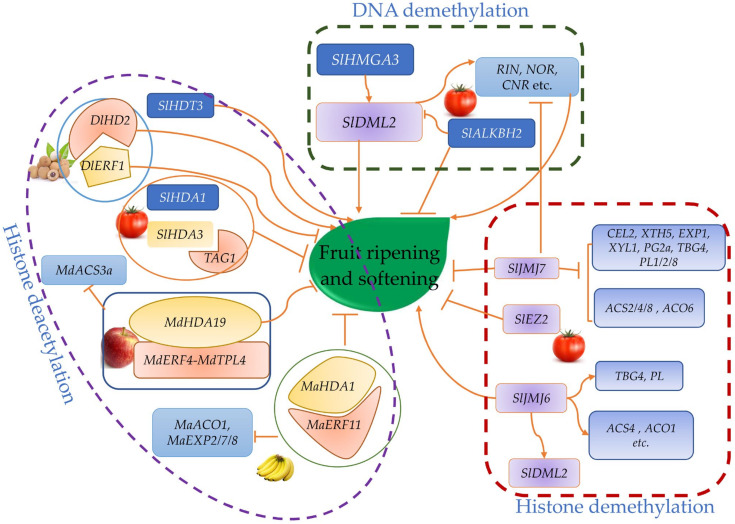
Role of epigenetic modifications in ripening and softening of fleshy fruits. The arrows indicate activation, whereas arrows with blunt ends indicate inhibition (see text).

In recent years, substantial evidence has emerged, supporting that epigenetic regulation is an important factor in controlling agronomic traits [141]. The epigenetic modification includes DNA methylation, post-translational histone modification, chromatin remodeling and so on. In addition to TFs and phytohormones, epigenetic regulation plays an essential role in fruit growth, development and ripening (Figure 3).

### 5.1. DNA Methylation Affects Fruit Softening

DNA methylation is a biological process by which methyl groups are added to the DNA molecule. Methylation can change the activity of a DNA segment without changing the sequence. When methyl groups are located in a gene promoter, DNA methylation typically acts to repress gene transcription [142]. During fruit development and ripening, the methylation and demethylation levels of genome occur dynamically. The researcher found that the reduction in methylation is associated with fruit ripening. Hadfield et al. reported that reduced DNA methylation of highly expressed genes in tomato fruit coincided with the onset of ripening [143]. However, the first evidence that DNA methylation affects tomato softening was a map-cloning of the gene at the *COLORLESS NON-RIPENING* (*CNR*) locus, a spontaneous mutation in *cnr*; the *cnr* mutant has hypermethylation in its promoter region, but its coding region is normal. The ripening, softening and carotenoid accumulation of the *cnr* mutant tomato fruit are significantly inhibited [144]. Genome-wide methylation analysis showed the reduction in methylation levels of genes related to fruit ripening and softening such as *ACO1*, *ACS2*, *PG*, *PMEU1*, *RIN*, *NOR*, *CNR* and *TAGL1* during the ripening of tomato fruits [145]. Later, Liu et al. [146] found that the degree of the promoter methylation of genes was related to their expression, while demethylation is catalyzed by demethylase such as DML, *SlDML2* in tomato, which is involved in the regulation of fruit ripening and softening. The knockdown or knockout of *SlDML2* up-regulates the expression of genes related to coloration, flavor synthesis, synthesis and signaling of ETH and cell wall hydrolysis, resulting in the delay of tomato fruit ripening and softening [147]. DNA methylation also can target *SlALKBH2*-encoding RNA demethylase to regulate the m^6^A methylation of mRNA. Delays in the fruit ripening of the *SlALKBH2* mutant may be a result of the feedback regulation of *SlDML2* stability [148]. Recent studies have shown that *SlHMGA3* encoding High-Mobility Group (HMG) A proteins can bind to the *SlDML2* promoter and activate its expression, resulting in the promoter DNA methylation of ripening-related TFs regulating ETH biosynthesis and signal transduction [149].

### 5.2. Histone Demethylation Modification during Fruit Softening

The post-translational modification of histones regulates gene expression by affecting chromatin structure. Reversible covalent modifications of histones include methylation, acetylation, phosphorylation and ubiquitination. *SlJMJ6* encoding a histone H3K27me2/3 demethylase in tomato was found to be a positive regulator of fruit ripening by directly activating the transcription of genes related to ripening such as genes for the synthesis of ETH (*ACS4*, *ACO1*, etc.) and genes related to fruit softening (*PL* and *TBG4*) to reduce their H3K27me3 levels; its overexpression accelerates tomato fruit ripening and softening [150]. Nevertheless, SlJMJ7, an H3K4 demethylase, is a critical negative regulator of fruit ripening in tomato. Overexpressing *SlJMJ7* represses the expression of target genes by removing the H3K4me3 of genes related to cell wall modification (*CEL2*, *XTH5*, *EXP1*, *XYL1*, *PG2a*, *TBG4* and *PL1*/*2*/*8*), ETH biosynthesis-related genes (*ACS2/4/8* and *ACO6*) and transcriptional regulators (*RIN*, *NOR* and *CNR*). SlJMJ7 also directly represses *SlDML2* expression by H3K4me3 demethylation during fruit ripening [22].

### 5.3. Roles of Histone Acetylation in Fruit Softening

Histone deacetylases (HDACs) also affect the expression of genes involved in tomato fruit development and ripening. For example, *SlHDA1* and *SlHDA3* encode the histone deacetylase, silencing SlHDA3 to induce the expression of genes related to ETH synthesis and cell wall metabolism, which accelerates fruit ripening and softening. [151,152]. Silencing *SlHDT3* gene-encoding histone deacetylase delays fruit softening by down-regulating the expression of genes related to fruit cell wall metabolism (*HEX*, *MAN*, *TBG4*, *XTH5* and *XYL*) [153]. PRC2 is responsible for the trimethylation of histone H3 at Lys27. Silencing the *SlEZ2* gene encoding the PRC2 protein Zeste enhancer (E(z)) accelerates fruit softening, [154,155].

Epigenetic modifications also are observed during the ripening and softening of other fleshy fruits. In strawberries, DNA methylation levels were reduced during its ripening. At the same time, DNA methyltransferase genes (*FvDRM1.3* and *FvDRM3.1*) were down-regulated, and genes related to photosynthesis and cell wall synthesis were inhibited [156]. Hu et al. [157] also found that *MdHDA19* promotes the production of ETH by H3K9 deacetylation in apples, and the silencing of *MdHDA19* resulted in decreased fruit firmness. *MaHDA1* gene-encoding histone deacetylase in bananas negatively regulates the expression of ripening-related genes during fruit ripening by interacting with TF MaERF11 [124]. The histone deacetylase 2-like gene (*DlHD2*) may interact with the ETH response factor-like gene (*DlERF1*) to regulate gene expressions related to fruit senescence [158].

## 6. Conclusions and Perspectives

Fruit softening is a complex process regulated by cell wall metabolism-related en-zymes, phytohormones, TFs and epigenetics, all four of which interact with each other and synergistically regulate fruit softening. This review summarizes recent advances on the effects of various regulatory factors on fruit softening. The cell wall has been consid-ered a major factor in fruit softening for a long period of time, where several proteins are coordinated and interdependent for cell wall metabolism and modification. As endogenous substances in the fruit, phytohormones play an important role in fruit development and ripening, and ETH and ABA are particularly involved in fruit softening after ripening. In addition, ETH interacts with other phytohormones such as IAA and ABA to regulate the expression of genes related to cell wall metabolism to regulate fruit softening. TFs regulate the expression of downstream target genes related to fruit ripening and softening. More importantly, the study of epigenetic modifications in fleshy fruits, epigenetic markers associated with fruit texture traits and the distribution of DNA methylation may identify important new targets for plant breeding and crop improvement. The epigenetic regulation of fruit ripening is an area that deserves intensive research, providing great potential for fruit quality improvement and breeding. However, fruit softening is more complex than expected, and more research is needed to fill the gaps. For example, the questions are about the role of secondary metabolites produced during fruit ripening in fruit softening and how they coordinate with fruit softening to obtain beneficial nutrients for humans. The contribution of cell wall modifications to fruit softening is not fully understood and the exact role of cell wall structural changes in fruit softening needs further elucidation. Interaction evidence between TFs and ripening-related proteins during fruit ripening is incomplete, especially for fruit softening-related effectors. The establishment of linkage networks between TFs and phytohormones by hormone-related regulatory factors needs to be enriched. Epigenetic alleles of epigenetic modifications need to be explored using techniques such as gene editing to obtain superior cultivars that do not affect postharvest fruit quality but extend fruit shelf life.

## Figures and Tables

**Table 1:** Regulation of cell wall metabolizing enzyme-related genes on the ripening and softening of fleshy fruits.

Cell Wall Degrading Enzyme		Function	Species	Encoding Genes	Reference(s)
PME (EC 3.1.1.11)		Removal of methyl groups from esterified pectin	Tomato (*Solanum lycopersicum* L.)	*PMEU1*, *PE1/2*	[43,46,47]
			Apricot (*Prunus armeniaca* L.)	*PaPME1*	
			Strawberry (*Fragaria vesca* L.)	*FvPME38*, *FvPME 39*	
PL (EC 4.2.2.2)		Eliminative cleavage of pectate, yielding oligosaccharides with 4-deoxy-α-D-mann-4-enuronosyl groups at their nonreducing ends	Tomato (*Solanum lycopersicum* L.)	*PL*	[7,48,49]
			Strawberry (*Fragaria vesca* L.)	*FvPLA*	
			Peach (*Prunus persica* *(L.) Batsch*)	*PpePL1*, *PpePL15*	
PG (EC 3.2.1.15)		Hydrolytic cleavage of α-l,4-galactouronosyl linkages in unesterified pectin	Tomato (*Solanum lycopersicum* L.)	*SlPG49*	[50,51,52,53]
			Strawberry (*Fragaria×ananassa* Duch.)	*FaPG1*	
			Peach (*Prunus persica* (L.) Batsch)	*PpPG21*, *PpPG22*	
			Grapevine (*Vitis vinifera* L.)	*VvPG1*, *VvPG2*	
			Banana (*Musa acuminata* Colla)	*MaPG3*, *MaPG4*	
			Pear (*Pyrus communis* L.)	*PcPGl*, *PcPG3*	
β-Gal (EC 3.2.1.23)		Removal of galactosyl residues increased from pectin	Tomato (*Solanum lycopersicum* L.)	*TBG4*	[55,56,57,58]
			Strawberry (*Fragaria × ananassa* Duch.)	*FaβGal4*	
			Peach (*Prunus persica* (L.) Batsch)	*PpBGAL2*, *PpBGAL16*, *PpBGAL10*	
XTH (EC 2.4.1.207)		Hydrolysis of xyloglucan or catalytic breakage of 1,4-β-D-glycosidic bond of xyloglucan and transfer of glycosyl groups	Apple (*Malus × domestica* Borkh.)	*MdXTHB*	[60,61,62,63]
			Strawberry (*Fragaria vesca* L.)	*FvXTH6*, *FvXTH9*	
			Persimmon (*Diospyros kaki* Thunb.)	*DkXTH6*, *DkXTH7*	
			Tomato (*Solanum lycopersicum* L.)	*SlXTH1*	
Expansin		Wall stress relaxation and irreversible wall extension	Tomato (*Solanum lycopersicum* L.)	*LeEXP1*	[67,69,70,71,72]
			Plum (*Prunus* spp.)	*PsEXPA10*	
			Strawberry (*Fragaria × ananassa* Duch.)	*FaEXPA2*, *FaEXPA5*	
			Banana (*Musa acuminata* Colla)	*MaEXP1*	
Other enzymes	Pectin acetylesterase (PAE)	Hydrolysis of acetyl esters of pectin, producing pectate, partially esterified pectin	Apple (*Malus × domestica* Borkh.)	*MdPAE10*	[73]
	endo-1,4-β-D-glucanohydrolase (EC 3.2.1.4)	Hydrolysis of soluble cellulose to produce reducing oligosaccharides	Strawberry (*Fragaria × ananassa* Duch.)	*FaEG1*	[74]

**Table 2:** TFs involved in the regulation of ripening and softening in fleshy fruits.

TF Types		Species	TFs Related to Fruit Ripening and Softening	Target Genes	Interacting Proteins	References
MADS-box		Tomato (*Solanum lycopersicum* L.)	RIN	*PG*, *TBG4*, *EXP1*, *CEL2*, etc.	FUL1/FUL2, TAGL1, SlMBP15, SlCMB1, NAC4, etc.	[112,113]
			SlMBP8	/	RIN	[111]
		Banana (*Musa acuminata* Colla)	MaMADS1, MaMADS2	/	/	[114]
		Peach (*Prunus persica* (L.) Batsch.)	PrupeSEP1	*PrupePG2*, *PrupePG3*	/	[115]
			PrupeFUL4	/	/	[116]
		Sweet cherry (*Prunus avium* L.)	PaMADS7	*PaPG1*	/	[117]
AP2/ERF		Tomato (*Solanum lycopersicum* L.)	AP2a	/	/	[119]
			LeERF1			[140]
			SlERF.B3	/	/	[120]
			SlERF.F12	*ACS2*, *ACS4*, *PG2a*, *PL*	TPL2, SlDHA1, SlDHA3	[121]
		Banana (*Musa acuminata* Colla)	MaERF11	*MaACO1*, *MaEXP2*, *MaEXP7*, *MaEXP8*	MaHDA1	[124]
		Peach (*Prunus persica* (L.) Batsch.)	PpERF4	*PpNCED2*, *PpNCED3*, *PpPG1*, *PpACO1*, *PpIAA1*	PpIAA1	[97]
			PpERF/ABR1	*PpPG*	/	[122]
		Papaya (*Carica papaya* L.)	CpERF9	*CpPME1/2*, *CpPG5*	/	[123]
		Apple (*Malus × domestica* Borkh.)	MdERF4	*MdERF3*	MdTPL4	[16]
NAC		Tomato (*Solanum lycopersicum* L.)	NOR-like1(SNAC4)	*SAPK3*, *SlCYP707A1*, *SlACO6*, *SlACS8*, *SlPG2a*, *SlPL*, *SlCEL2*, *SlEXP1*	SAPK3, SlPYL9, SlACS2, SlACO1	[125,126]
			NOR	*SlACS2*, *SlGgpps2*, *SlPL*	/	[127]
			SlNAC1	*SlPSY1*, *SlACS2*, *SlACO1*	/	[128]
		Melon (*Cucumis melo* L.)	CmNAC-NOR	*CmACS5, CmNCED3*	/	[129]
		Banana (*Musa acuminata* L.)	MaNAC1	*MaERF11*, *MaXB3*	/	[130]
			MaNAC2	*MaERF11*, *MaXB3*	MaXB3	
		Chilean strawberry (*Fragaria chiloensis* (L.) Duch.)	FcNAC1	*FcPL*	/	[131]
		Strawberry (*Fragaria × ananassa* Duch.)	FaRIF(FaNAC035)	/	/	[132]
MYB		Tomato (*Solanum lycopersicum* L.)	SlMYB70	*SlACS2, SlACO3*	/	[89]
			SlMYB75	*SlFSR*	/	[134]
		Wild strawberry (*Fragaria vesca* L.)	FvMYB79	*FvPME38*	/	[135]
BZR		Tomato (*Solanum lycopersicum* L.)	SlBES1	*PMEU1*	/	[20]
		Banana (*Musa acuminata* Colla)	MaBZR1/2	*MaEXP2*, *MaPL2*, *MaXET5*	MaMPK14	[136]
Others	GRAS	Tomato (*Solanum lycopersicum* L.)	SlFSR	/	/	[17]
	ARF	Papaya (*Carica papaya* L.)	CpARF2	/	CpEIL1	[137]
	LOB	Tomato (*Solanum lycopersicum* L.)	SlLOB1	*EXP1*, *CEL2*, *XY*, *AGP2*, *TBL*, *E6*, *PL*	/	[15]
	WRKY	Wild strawberry (*Fragaria vesca* L.)	FvWRKY48	*FvPLA*	/	[48]
	bZIP	Banana (*Musa* spp.)	MabZIP74	*MaACO1*/*4*	MaMAPK11-3	[138]
	bHLH	Banana (*Musa* spp.)	MaTCP5, MaTCP19,	*MaXTH10*/*11*	MaTCP20	[139]
			MaTCP20	*MaXTH10*/*11*	MaTCP5, MaTCP19,	

## Data Availability

The original contributions presented in the study are included in the article, further inquiries can be directed to the corresponding author.
